# Neural Univariate Activity and Multivariate Pattern in the Posterior Superior Temporal Sulcus Differentially Encode Facial Expression and Identity

**DOI:** 10.1038/srep23427

**Published:** 2016-03-21

**Authors:** Zetian Yang, Zonglei Zhen, Lijie Huang, Xiang-zhen Kong, Xu Wang, Yiying Song, Jia Liu

**Affiliations:** 1 State Key Laboratory of Cognitive Neuroscience and Learning & IDG/McGovern Institute for Brain Research, Beijing Normal University, Beijing, 100875, China; 2 The Rockefeller University, 1230 York Avenue, New York, NY 10065, USA; 3 Beijing Key Laboratory of Applied Experimental Psychology, School of Psychology, Beijing Normal University, Beijing, 100875, China

## Abstract

Faces contain a variety of information such as one’s identity and expression. One prevailing model suggests a functional division of labor in processing faces that different aspects of facial information are processed in anatomically separated and functionally encapsulated brain regions. Here, we demonstrate that facial identity and expression can be processed in the same region, yet with different neural coding strategies. To this end, we employed functional magnetic resonance imaging to examine two types of coding schemes, namely univariate activity and multivariate pattern, in the posterior superior temporal cortex (pSTS) - a face-selective region that is traditionally viewed as being specialized for processing facial expression. With the individual difference approach, we found that participants with higher overall face selectivity in the right pSTS were better at differentiating facial expressions measured outside of the scanner. In contrast, individuals whose spatial pattern for faces in the right pSTS was less similar to that for objects were more accurate in identifying previously presented faces. The double dissociation of behavioral relevance between overall neural activity and spatial neural pattern suggests that the functional-division-of-labor model on face processing is over-simplified, and that coding strategies shall be incorporated in a revised model.

One face is worth a thousand words. A face speaks in a fraction of second an individual’s identity, mood, and direction of attention, among other socially important information. Neuroimaging studies have identified a dedicated face processing system composed of multiple face-selective regions (for a review see^1^. However, while the location and organization of these regions is well established^2^,^3^, it is still unclear how different aspects of facial information are represented and processed in these regions^4^,^5^,^6^.

One region that has received special attention is the face-selective posterior superior temporal sulcus (pSTS). Studies have long confirmed the role of the pSTS in processing dynamic facial information such as facial expression^7^,^8^,^9^,^10^. However, recent studies have added to this view by demonstrating the region’s sensitivity to static facial information such as facial identity^6^,^11^,^12^,^13^. The new finding that the pSTS is involved in the processing of both facial identity and expression poses a computational difficulty in that two distinct types of information being represented within the same brain region^5^,^14^. More specifically, neural representations of facial expressions in the pSTS must possess the ability to consistently vary according to changes in facial muscles. In contrast, in order to reliably recognize an individual’s identity, the neural representation of identity must be relatively invariant to facial changes; otherwise, any changes may invoke the perception of a new identity^14^. To reconcile these two extreme demands, we speculated that the pSTS may adopt qualitatively different neural coding strategies to represent facial expression and identity.

Functional magnetic resonance imaging (fMRI) studies have revealed that brain regions can represent information in at least two ways, namely via univariate activity (i.e., magnitude of neural activity averaged across voxels in a region) and multivariate pattern (i.e., spatial pattern of neural activity among voxels within a region). Univariate activity in a region, which seeks commonality among activities of voxels, recognizes the voxels as homogeneous, and thus can be seen as a localized code. In contrast, multivariate pattern examines the spatial distribution of activity among voxels, and reflects more of a distributed code^15^,^16^.

In the present study, we hypothesized that facial expression and identity may be both represented in the pSTS yet with localized and distributed codes respectively. To test this, we scanned a large cohort of participants (N = 202) while they passively viewed faces and non-face objects, and then calculated the overall face selectivity (Z score from the contrast of faces versus objects averaged across voxels) and between-category pattern dissimilarity (one minus the correlation coefficient between the spatial pattern for faces and that for objects). Outside of the scanner, we used an old/new recognition task^17^,^18^ to measure the participants’ ability to recognize facial identity, and the Reading the Mind in the Eyes Test (henceforth, Eyes Test^19^) to measure their ability to recognize facial expression. Finally, we examined the relationship between the coding strategies of the pSTS and behavioral performance in the recognition of facial identity and expression.

## Results

**Figure 1:**
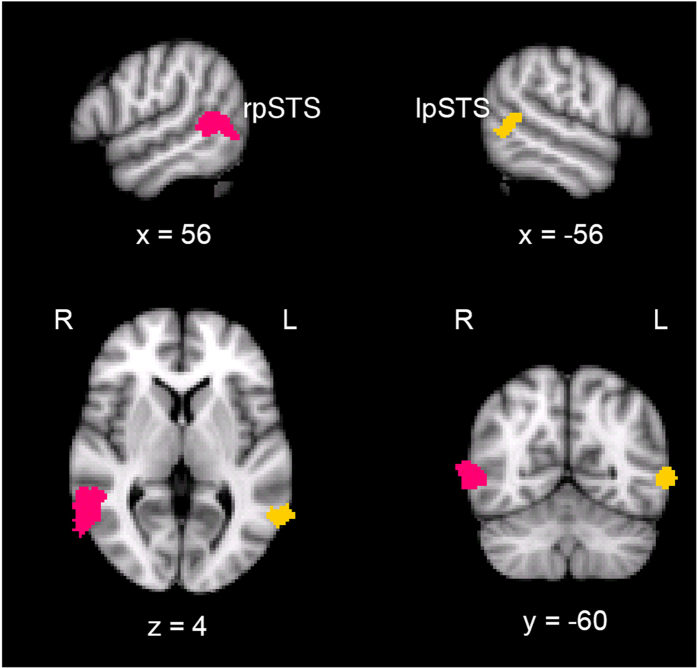
The right pSTS (magenta) and left pSTS (gold) in a typical subject.

Using the contrast corresponding to neural responses to faces versus objects, the pSTS was bilaterally identified in each participant. Specifically, the pSTS was defined as a set of contiguous voxels around the posterior part of the STS. Of the 191 participants with both neuroimaging data and behavioral performances, the right pSTS was successfully identified in 165 (86%) participants, whereas the left pSTS was identified in 133 (70%) participants (for MNI coordinates of peak voxel and size, see Table 1). The left pSTS being identified in fewer participants is consistent with previous studies that have reported that the right pSTS shows more robust activation to faces than its left counterpart (e.g.^20^). Figure 1 shows the location of the right and left pSTS in a typical participant.

Having identified the pSTS, we then investigated the relationship between two neural codes of the pSTS and participants’ behavioral performance in facial identity and expression recognition. One neural code was measured by the overall face selectivity, indexed by the Z score from the contrast of faces versus objects averaged across voxels in the pSTS; the other neural code was calculated by between-category pattern dissimilarity, which was one minus the correlation coefficient between the spatial pattern for faces and that for objects. For behavioral measures, we used an old/new face recognition task (i.e., participants were asked to judge whether a face was presented previously) to test identity recognition ability, and the Eyes Test to measure expression recognition ability (for summary of behavioral performance, see Table 2).

### Overall face selectivity is associated with the recognition of facial expression

**Figure 2:**
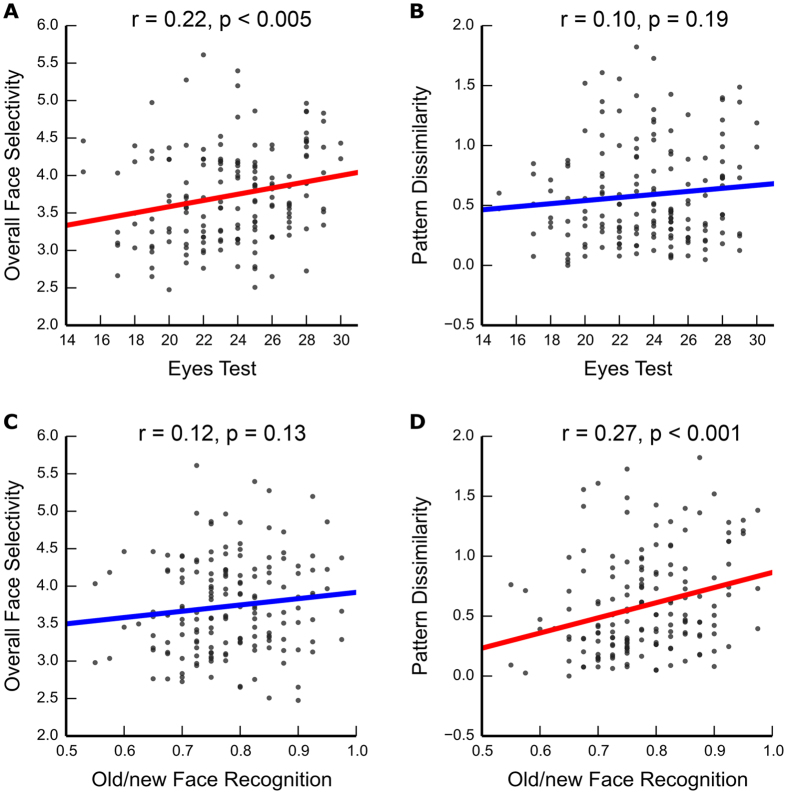
Correlations between the neural codes in the right pSTS and behavioral performances in facial identity and expression recognition tasks (N = 165). Scatter plots are shown between (**A**) expression recognition and overall face selectivity, (**B**) expression recognition and between-category pattern dissimilarity, (**C**) identity recognition and overall face selectivity, and (**D**) identity recognition and between-category pattern dissimilarity.

In the right pSTS, we found that participants’ behavioral performance in the Eyes Test was correlated with their overall face selectivity (Pearson’s r = 0.22, p = 0.004; Fig. 2A), but not with pattern dissimilarity (Pearson’s r = 0.10, p = 0.19; Fig. 2B). Besides, statistical test showed that there was an insignificant trend toward the correlation between expression recognition and face selectivity being larger than the correlation between expression recognition and pattern dissimilarity (Steiger’s Z = 1.36, one-tail p = 0.09). Using the prediction analysis based on cross-validation, we confirmed that expression recognition could be predicted by the overall face selectivity (r(prediction, observation) = 0.21, p = 0.002), but not pattern dissimilarity (r(prediction, observation) = 0.036, p = 0.18). On the other hand, no correlation was found between expression recognition and the two neural codes in the left pSTS (overall selectivity: Pearson’s r = 0.04, p = 0.61; r(prediction, observation) = 0.001, p = 0.31; pattern dissimilarity: Pearson’s r = −0.04, p = 0.66; r(prediction, observation) = −0.05, p = 0.45).

To control for confounding factors that could account for the correlation in the right pSTS, we conducted a multiple regression analysis with expression recognition as a dependent variable and overall face selectivity as a predictive variable. Gender was added as the first covariate, as a significant gender difference was shown in expression recognition (Table 2). Further, because of a positive correlation between overall face selectivity and pattern dissimilarity in the right pSTS (Pearson’s r = 0.34, p < 0.0001), pattern dissimilarity was added as a covariate to examine whether the association between overall face selectivity and expression recognition was independent from the contribution of pattern dissimilarity.

The regression analysis revealed a significant association between expression recognition and overall face selectivity (β = 0.86, p = 0.047; Table 3) after controlling for variance from gender and pattern dissimilarity. Gender was also found to be independently correlated with expression recognition (β = 1.66, p = 0.002; Table 3). Moreover, no significant contribution from pattern dissimilarity was found. Taken together, our results suggest that overall face selectivity, not pattern dissimilarity, independently predicts facial expression recognition in the right pSTS.

### Pattern dissimilarity is associated with the recognition of facial identity

Again in the right pSTS, we found that behavioral performance in recognizing facial identity was correlated with between-category pattern dissimilarity (Pearson’s r = 0.27, p < 0.001; Fig. 2D), not with overall face selectivity (Pearson’s r = 0.12, p = 0.13; Fig. 2C). Moreover, the correlation between identity recognition and pattern dissimilarity was significantly larger than the correlation between identity recognition and face selectivity (Steiger’s Z = 1.71, one-tail p = 0.04). A prediction analysis confirmed that identity recognition could be predicted by pattern dissimilarity (r(prediction, observation) = 0.25, p = 0.0002), not by overall face selectivity (r(prediction, observation) = 0.070, p = 0.12). Similarly, identity recognition was not correlated with either neural codes of the left pSTS (pattern dissimilarity: Pearson’s r = 0.03, p = 0.70; r(prediction, observation) = −0.03, p = 0.40; overall selectivity: Pearson’s r = 0.075, p = 0.40; r(prediction, observation) = 0.01, p = 0.29).

To examine whether the association that we found was face-specific, we conducted a multiple regression analysis with facial identity recognition as a dependent variable and pattern dissimilarity as an independent variable. Three covariates were added to control for unwanted sources of variance. The first covariate was behavioral performance in recognizing objects, more specifically, the accuracy of recognizing flowers in the old/new recognition task. This covariate was used to control for domain-general cognitive abilities such as visual-discrimination abilities, visual short-term memory, and attention. The second covariate added was gender, as previous studies have suggested that females are better than males at face recognition^21^. However, it should be noted that we did not find a significant difference between females and males in the old/new face recognition task (Table 2). Nevertheless, we added gender as a covariate controlling for variance contributed by gender difference. The final covariate was overall face selectivity because of its association with between-category pattern dissimilarity.

The multiple regression analysis revealed that there was a significant association between pattern dissimilarity and facial identity recognition (β = 0.049, p = 0.006; Table 3), as well as a significant correlation between object recognition and facial identity recognition (β = 0.211, p = 0.01; Table 3). No other significant associations were found. Taken together, these findings suggest that it is the pattern dissimilarity, not overall face selectivity, that accounts for the unique variance of facial identity recognition.

Another possible confounding factor contributing to the association between pattern dissimilarity and identity recognition is the noise in neural activity. Indeed, if neural activity is noisier, the variance of the activity is larger, which in turn leads to higher dissimilarity between neural patterns. Therefore, it might be the noise, rather than the dissimilarity of neural responses to different categories, that drives the association between identity recognition and between-category pattern dissimilarity. To rule out this possibility, we estimated the amount of noise in neural activity by calculating the pattern dissimilarity between neural patterns of independent runs to the same category (i.e., test-retest reliability). That is, for each participant, we extracted two neural patterns for the face category from the first and second run respectively, and then calculated the within-category pattern dissimilarity as an index for the amount of noise in neural activity. We found no significant association between the within-category pattern dissimilarity and the behavioral performance in recognizing faces (Pearson’s r = 0.10, p = 0.19; r(prediction, observation) = 0.06, p = 0.13). Further multiple regression analysis with the between-category pattern dissimilarity as a predictive variable and the within-category pattern dissimilarity (i.e., noise) as a control variable showed that the between-category pattern dissimilarity predicted facial identity recognition after the noise was controlled for (β_pattern dissimilarity_ = 0.058, p = 0.001; β_noise_ = 0.0075, p = 0.71). Thus, it is the distinction of the pattern in neural responses to different categories, not the noise in neural activity, which accounts for the association.

In summary, in the right pSTS we found a double dissociation between overall face selectivity and pattern dissimilarity as distinct neural predictors for facial expression and identity recognition, respectively.

## Discussion

The present study investigated how qualitatively different aspects of facial information (i.e., identity and expression), are both represented in the face-selective pSTS. By comparing the behavioral relevance of univariate activity and multivariate pattern with behavioral performance in facial identity and expression recognition, we found a double dissociation in the right pSTS. That is, the overall face selectivity of the right pSTS could predict facial expression recognition, whereas the between-category pattern dissimilarity was associated with facial identity recognition. The double dissociation suggests that the right pSTS takes up different coding strategies to independently process facial expression and identity.

The finding that overall face selectivity of the right pSTS could predict facial expression recognition ability is consistent with the view that the pSTS plays a critical role in processing facial expression^4^,^7^. Empirical studies have revealed the pSTS showing higher response to emotional facial expressions than to neutral faces^8^,^22^ and had causal role in facial expression discrimination^10^. Our finding adds to these studies by providing the first evidence that face-selective responses in the right pSTS were correlated with individual differences in ability to recognize facial expressions. Moreover, this correlation cannot be accounted for by a general cognitive process such as IQ, task engagement, or visual discrimination, or by general characteristics of the participants, such as age or health. That is because we did not observe any correlations between the other neural measure (i.e., pattern dissimilarity) and expression recognition. One possibility that could account for the association is that overall face selectivity in the right pSTS partly reflects the amount of attention allocated to expression-related information. This hypothesis is consistent with studies that have reported enhanced activity in the right pSTS when participants were asked to selectively pay attention to facial expression rather than to face identity per se^22^. As we used a passive view paradigm during the scan, the higher activity in the pSTS of participants with better expression recognition ability might reflect that they were more likely attracted to facial expression in a bottom-up fashion. This could in turn lead to their better performance at discriminating subtle differences between expressions.

The association that we found between pattern dissimilarity and the recognition of facial identity is less clear. One prevailing model of face perception proposes that identity and expression are processed by two separate pathways^4^,^14^. More specifically, the model suggests that facial identity is mainly processed in the ventral pathway including the fusiform face area (FFA^23^), while facial expression is processed in the lateral pathway composed of the pSTS. This view of complete independence between the processing of facial identity and expression has been challenged by recent findings connecting the pSTS with facial identity. First, fMRI adaptation studies have revealed that the right pSTS is sensitive to changes in facial identity^12^,^13^,^24^. Second, a neuropsychological study reports that a patient with a lesion in the face-selective pSTS exhibits difficulty in discriminating facial identity^25^. Third, using transcranial magnetic stimulation, Pitcher *et al.*^6^ report that the right pSTS shows a reduced response to static faces when disrupting the right occipital face area (OFA^26^), suggesting that static facial information may reach the pSTS, possibly relayed by the OFA. Our study supplements these findings, in that between-category pattern dissimilarity in the right pSTS was associated with facial identity recognition ability, demonstrating the efficacy of pattern dissimilarity as a neural correlate of object recognition ability^27^. Note that although multivariate patterns show a higher sensitivity than univariate activation in encoding information^15^,^28^, it does not necessarily promise that they are a better neural predictor for behavioral performance (i.e., predictability). Consistent with this intuition, our data have shown that the pattern performed worse than the overall face selectivity in predicting behavioral performance in differentiating facial expressions.

Although evidence suggests that the pSTS does process facial identity, the underlying mechanism remains unclear. Our study sheds new light on how the pSTS processes both facial expression and identity via different neural coding strategies. One possibility is that there are two distinct populations of neurons in the right pSTS that are separately sensitive to facial identity and expression, with the identity-sensitive neurons interspersed in the (more abundant) expression-sensitive neurons^8^. Thus, the overall face selectivity of fMRI data mainly reflects the activity of expression-sensitive neurons but largely ignores the activity of identity-sensitive neurons. On the other hand, the multivariate pattern is able to pick up the subtle activity of identity-selective neurons spread within the region but largely ignores the overall level of responses driven by expression-sensitive neurons. Another possibility is that there is only one population of neurons, but with identity- and expression-related information being encoded by different aspects of neuronal activity. If this were the case, then expression would be largely coded by the average firing rate of neurons, whereas facial identity by the spatial arrangement of the neuron population’s firing rates. Indeed, this hypothesis would explain why some neurons in the STS are sensitive to both identity and dynamic facial features^29^,^30^. Future neurophysiological studies are needed to address these and other hypotheses in order to illuminate the neural mechanisms underlying our findings.

The finding that the brain-behavior association was only observed in the right but not left pSTS is consistent with the fact that cortical face processing is largely right lateralized. For example, behavioral studies show a better retention and recognition of faces presented in the left visual field (e.g.^31^,^32^,^33^,^34^), and face-selective regions in right hemisphere are larger in size^35^,^36^ and stronger in neural activation^3^. In addition, acquired prosopagnosia is rarely associated with unilateral lesion in left occipitotemporal cortex^37^,^38^; it is instead more common with unilateral right or bilateral lesions^39^,^40^. The right lateralization of cortical face processing is particularly true for the pSTS. For example, the left pSTS is less reliable to be localized^23^,^41^ and has a smaller volume and weaker responses^3^,^42^. In addition, TMS studies show a causal link of the right pSTS, but not the left pSTS, in facial processing^6^. Therefore, it is not surprising that we failed to observe the brain-behavior association in the left pSTS, possibly because of the lack of sufficient information in the left pSTS for facial expressions.

In the current study, we used the pattern dissimilarity as a multivariate measure of the neural activity. Another popular multivariate measure is the classification accuracy (i.e. using multivariate patterns to classify facial identity or expression). However, our design prevented us from such classification analyses for two reasons. First, the fMRI scan and the behavioral test were conducted separately with different stimuli and tasks. This design, on one hand, enabled us to generalize the brain-behavior association across stimuli and tasks, but, on the other hand, prevented us from estimating neural activity for each stimulus. Future studies with proper designs may use classification accuracy in the pSTS as a multivariate measure to predict behavioral performance in face recognition.

In summary, our study has revealed that facial expression and identity recognition are differentially related to overall face selectivity and between-category pattern dissimilarity in the right pSTS. Our results also suggest that the brain processes facial information in a way that is much more complicated than simply separating identity and expression into two visual streams. We thus invite future studies to further resolve the intricacies of the neural representations and mechanisms underlying facial recognition.

## Methods

### Participants

Two hundred and two students (age range: 18–23 years; 124 females) were recruited from Beijing Normal University. All participants had normal or corrected-to-normal visual acuity. This study is part of our ongoing project to explore associations between brain imaging, cognitive function, and genetics^3^,^18^. Data that were irrelevant to the scope of this study are not reported here. All experiments were performed in accordance with the relevant guidelines and regulations of Beijing Normal University’s Institutional Review Board (Human Subjects Division), which approved all the experimental protocol and procedures. Written informed consent was obtained for every participant in the study.

### Experimental Procedure

Our experiments comprised of two parts: fMRI scanning and behavioral testing. The fMRI data were collected while participants passively viewed short video segments of different object categories. The region of interest (ROI) approach was used to bilaterally define the face-selective pSTS in every participant^3^. Then, the overall face selectivity and between-category pattern dissimilarity were calculated from the blood-oxygen-level dependent (BOLD) activity of the subject-specific pSTS.

Outside of the scanner, the same participants were asked to partake in two behavioral tasks aimed to test their abilities in facial expression and identity recognition. The ability of participants to recognize facial expression was measured by the Eyes Test, while participants’ identity recognition ability was measured by an old/new recognition task. We then investigated how expression and identity recognition related to overall face selectivity and between-category pattern dissimilarity in the pSTS. Though we used the same functional data for ROI definition and neural-activity measurement, this unlikely led to the problem of double dipping^43^ for two reasons. Firstly, our ROI-definition method was independent of participants’ behavioral performance; therefore, the neural measures and behavioral data used for correlation analyses were independent. Secondly, biases introduced by double dipping mainly exaggerate the mean values of the neural measures (e.g. the magnitude of activation tending to be larger); however, such biases have little impact on the variance of the neural measures across participants (i.e., individual differences), which the correlation analyses were based on.

All participants took part in in the fMRI scans. Two hundreds of the participants participated in the old/new recognition task, while 194 of the participants participated in the Eyes Test. Near-chance performances were found for two participants in the old/new recognition task and one participant in the Eyes Test. These three participants were thus excluded from further analyses, which were based on behavioral and fMRI data collected from 191 participants.

### fMRI Scanning

fMRI data were acquired on a SIEMENS TRIO 3T scanner at the Imaging Center for Brain Research, Beijing Normal University. Participants were instructed to lay in a supine position, with their heads snugly fixed with foam pads to minimize head movement. Functional images were collected using a gradient-echo EPI sequence in 3.1 × 3.1 × 4.8 mm voxels (repetition time (TR) = 2000 ms; echo time (TE) = 30 ms; flip angle = 90°; slices = 30). High-resolution structural images were collected using a 3D T1-weighted magnetization prepared rapid gradient-echo (MP-RAGE) sequence in 1.3 × 1.3 × 1 mm voxels (TR = 2500 ms; TE = 3.39 ms; flip angle = 10°; slices = 176).

Subjects participated in three runs during which 18-s blocks were presented. Each block comprised of six 3-s movie clips of faces, objects, scenes, or scrambled objects without interstimulus intervals (ISI)^3^,^20^. The movie clips of faces were recorded from a group of children dancing and playing. In each of these video clips, the same face showed continuous changes, exhibiting different expressions, eye gazes, and view angles. Moving objects such as rolling balls and natural sceneries were shot to make the object and scene movies, respectively. Finally, scrambled object movies were made by firstly dividing the object movies into small rectangles and then randomly rearranging the location of each rectangle. Each run lasted for 198 s, and consisted of 11 blocks, among which two groups of consecutive stimulus blocks were sandwiched by three fixation blocks. One block of each category was presented in the stimulus groups. In the fixation block, six full-screen colors were presented for 3 s each. During the scan, participants were asked to watch the movies but not to perform any overt tasks.

### fMRI Data Analysis

Functional data were processed using FEAT^44^ from FMRIB’s Software Library (FSL, http://www.fmrib.ox.ac.uk/fsl). The preprocessing steps included the following: motion correction, grand-mean intensity normalization, spatial smoothing with a Gaussian kernel (6 mm full width at half maximum), and temporal high-pass filtering. For each participant, the voxel time series were fit by a general linear model, with each condition modeled by a boxcar convolved with a gamma hemodynamic response function. In addition, the temporal derivatives of the convolved boxcars, as well as six parameters from the motion correction, were added to the model as covariates. Finally, all participants’ statistical maps were normalized to the MNI-152 template and resampled at 2 × 2 × 2 mm resolution.

### ROI Definition and Analyses

For each participant and hemisphere, the pSTS was defined as a set of contiguous voxels that showed a significantly higher response to faces than to objects (p < 10^−2^, uncorrected) around the posterior part of the superior temporal sulcus. Specifically, the individual activation image from the contrast of faces versus objects was first thresholded at Z > 2.3 (p < 0.01, uncorrected), and then the pSTS was delineated via a semiautomatic approach. For more details on the definition procedure, see^3^. Two measures of the localized and distributed coding strategies were then calculated separately; namely, overall face selectivity and between-category pattern dissimilarity. Overall face selectivity was based on univariate neural activity and was calculated by averaging Z scores from the contrast of faces versus objects across all voxels within the pSTS. The between-category pattern dissimilarity was calculated based on multivariate pattern analysis and was defined as one minus the correlation between the spatial patterns of responses for faces and objects in the pSTS. Beta weights for faces and objects were used as response strengths, from which mean responses across all categories had been subtracted before calculating correlation^45^. Lastly, it should be noted that both overall face selectivity and between-category pattern dissimilarity carry information about the strength of a region’s response to its preferred stimulus category; thus, the larger the value, the stronger a region activates to that category.

### Behavioral Tests

#### Old/New Recognition Task

**Figure 3:**
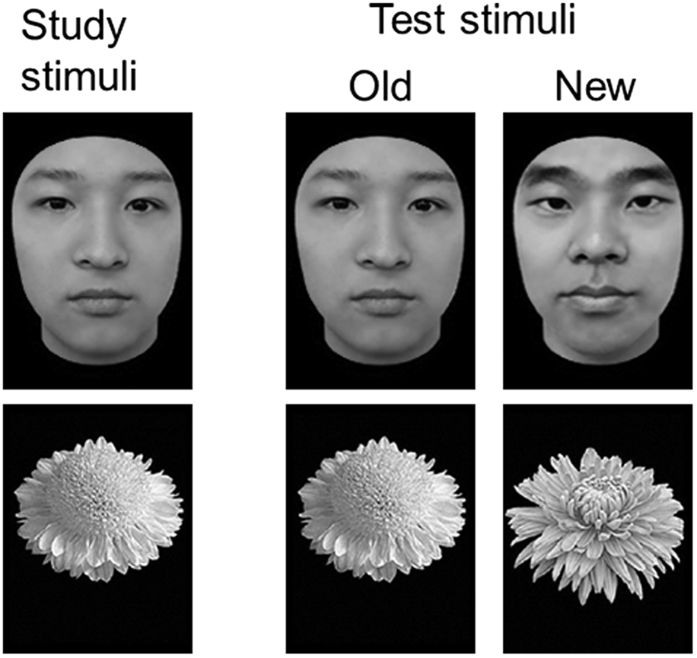
Example stimuli of the old/new face and flower recognition task. Participants firstly studied a set of faces and flowers, which were then mixed with a set of new stimuli, and the participants were asked to indicate whether each picture had been studied before (see also Wang *et al.*^17^; Huang *et al.*^18^). Face stimuli shown in the figure are for the display purpose only, which were not present in the test.

Forty images of faces and 40 images of flowers were used in this task (Fig. 3). The face images were gray-scale pictures of adult Chinese faces, of which the external contours had been removed to leave a roughly oval shape without hair. The flower images were gray-scale pictures of commonly seen flowers with their leaves removed on a blank background. The task comprised of a face block and a flower block. Each block consisted of two segments: a study segment and a test segment. In the study segment, 20 images of an object category were shown twice, with each image lasting for 1 s, and an ISI of 0.5 s. In the test segment, the 20 studied images and 20 new images of the same category were shown in random order. On presentation of each image, participants were asked to determine whether the image had been presented in the study segment. Both face and flower blocks were counterbalanced across participants. For each category, accuracy was computed by summing all correct responses and converting to a percentage score.

#### Reading the Mind in the Eyes Test

The revised version of the Eyes Test consists of 36 gray-scale photographs of the area of the eyes, presenting subtle affective expressions. Participants were instructed to choose which of four words best described the emotion or mental state of the person in the photograph. The task thus involved the recognition of facial expression and the attribution of mental state based on that expression. Individual scores were calculated as the total number of correct answers, with the maximum total score being 36.

To make the test suitable for Chinese participants, several modifications were carried out. First, the English words depicting facial expressions were translated into Chinese. Second, the answer to each expression was revised to reflect culture difference. Based on the principle of setting target words for the Eyes Test^46^, we calculated the percentages of participants choosing each word for each expression, and if the percentage for a foil word was higher than 50%, the foil word was then set as the target word for the expression. Two samples of college students were recruited to participate in the Eyes Tests for this purpose. The first sample consisted of 286 students (mean age = 21.53, SD = 1.00, 154 female), and the second sample consisted of 268 students (mean age = 22.15, SD = 0.83, 162 female). The first sample participated only in the behavioral test, while the second sample participated in both the behavioral tests and MRI scans (i.e., the above-mentioned participants who were qualified for analyses in the current study were from this sample). Of all 36 expressions in the original test, only expression 17 met our criterion of modification. Specifically, 66.8% of the first sample and 69.3% of the second sample judged the expression as “affectionate” (a foil word in the original test), and only 27.9% of the first sample and 24.6% of the second sample judged the expression as “doubtful” (the original target word). As a result, the target word of the 17th expression was changed from “doubtful” to “affectionate”.

### Correlation and Prediction Analysis

We used a correlation analysis to test the association between measures of neural coding strategies and behavioral performance. As correlation is prone to over-fitting and lacks predictive validity, a balanced fourfold cross-validation method was used to validate the predictability of behavioral performance from the neural coding measures. To this end, a linear regression model was used in which the two behavioral performances were considered as dependent variables and the two neural measures were considered as independent variables. For each pair of dependent and independent variables, the dataset was divided into four folds under the restriction that there were no significant differences between the distributions of the data. For each fold of data, a linear regression model was estimated using data from the other three folds, and was then used to predict the data in the unused fold. After data from all folds had been predicted, the correlation between the predicted data and the actual observed data, r(prediction, observation), was calculated to measure the overall predictability of the observed data. The statistical significance of r(prediction, observation) was calculated by a nonparametric randomization approach. The data of the independent variable were randomly shuffled and an r_n_(prediction, observation) was calculated based on the shuffled independent variable and the original dependent variable. This procedure was repeated 5000 times to estimate the null distribution of r(prediction, observation). Finally, the significance of r(prediction, observation) was calculated as one minus the percentile of the true r(prediction, observation) among the null distribution.

## Additional Information

**How to cite this article**: Yang, Z. *et al.* Neural Univariate Activity and Multivariate Pattern in the Posterior Superior Temporal Sulcus Differentially Encode Facial Expression and Identity. *Sci. Rep.* **6**, 23427; doi: 10.1038/srep23427 (2016).

## Figures and Tables

**Table 1:** MNI coordinates of peak and ROI sizes averaged across participants (mean ± SD).

Region	MNI coordinates			Size (voxels)
	x	y	z	
Right pSTS	55 ± 7	−59 ± 6	7 ± 7	445 ± 376
Left pSTS	−57 ± 6	−62 ± 5	9 ± 6	291 ± 288

pSTS: posterior superior temporal sulcus.

**Table 2:** Mean and standard deviation of behavioral scores with gender difference.

Behavioral tests	Score	Gender difference		
	mean ± SD	t score	p	Cohen’s d
Old/new face	0.78 ± 0.09	1.41	0.16	0.03
Old/new flower	0.81 ± 0.08	0.8	0.43	0.12
Eyes test	24 ± 3	3.59	0.0004	0.58

**Table 3:** Multiple regression with behavioral performances as dependent variables and neural coding measures as independent variables.

Behavioral performance and predictor	β	SE β	Standardized β	p
Eyes Test				
Pattern dissimilarity	0.35	0.64	0.044	0.58
Overall Face selectivity	0.86	0.43	0.16	0.047
Gender	1.66	0.53	0.24	0.002
Old/new face recognition				
Pattern dissimilarity	0.049	0.017	0.23	0.006
Overall Face selectivity	0.0046	0.012	0.032	0.69
Flower recognition	0.21	0.083	0.19	0.011
Gender	0.016	0.014	0.086	0.26
